# The Potential of Virgin Olive Oil from cv. Chondrolia Chalkidikis and Chalkidiki (Greece) to Bear Health Claims according to the European Legislation

**DOI:** 10.3390/molecules26113184

**Published:** 2021-05-26

**Authors:** Aspasia Mastralexi, Maria Z. Tsimidou

**Affiliations:** 1 Laboratory of Food Chemistry and Technology, School of Chemistry, Aristotle University of Thessaloniki (AUTH), 54124 Thessaloniki, Greece; aspamastralexi@chem.auth.gr; 2 Natural Products Research Center of Excellence (NatPro-AUTH), Center for Interdisciplinary Research and Innovation (CIRI-AUTH), 57001 Thessaloniki, Greece

**Keywords:** virgin olive oil, Chondrolia Chalkidikis cultivar, Chalkidiki cultivar, health claims, oleic acid, vitamin E, olive oil phenols, maturity index, total hydroxytyrosol and tyrosol content

## Abstract

The European food legislation authorizes the use of certain health claims based on a scientific basis. This study aimed to evaluate the fatty acid, tocopherol, and polar phenol composition of virgin olive oil (VOO) from cv. Chondrolia Chalkidikis and Chalkidiki regarding the fulfillment of official requirements for the health claims of ‘oleic acid’, ‘vitamin E’, and ‘olive oil polyphenols’. The examination of representative industrial VOOs from 15 olive mills of the Chalkidiki regional unit showed that the two cultivars yield oils contained the necessary concentrations of the responsible bioactive compounds. This evidence was further substantiated by a four harvest study whereby olives from different maturity stages were sampled from three olive groves. Oils were extracted at a laboratory scale and examined for their content in the above-mentioned three categories of constituents. Oils produced at industrial scale from olives harvested on the ‘technological optimum’ stage according to the olive grove proprietor were also analyzed. Extra virgin olive oil of the studied cultivars can safely bear the generic claims for ‘oleic acid’ and ‘vitamin E’. The cultivars present great potential regarding the total hydroxytyrosol and tyrosol content of the extracted oil required to attain the third health claim that may be influenced negatively by manufacturing practices.

## 1. Introduction

The concern of consumers toward healthiness is continuously on the rise. Recent reports indicate that even the COVID-19 (COronaVIrus Disease-2019) pandemic experience had a positive impact as consumer behavior and agro-food markets shifted toward healthier food choices and more sustainable supply and production patterns [1].

On this basis, authorized nutrition and health claims may represent a key marketing tool to inform and guide consumers about the health benefits of the intrinsic constituents of a product. VOO is recognized by the scientific community as a functional fat due to beneficial effects on human health attributed to a variety of bioactive compounds [2]. Except from the high concentration of the monounsaturated fatty acids (MUFA) oleic acid (C18:1), VOO is characterized by a balanced ratio in α-tocopherol (α-T) vs polyunsaturated fatty acids (PUFA) content and the presence of various categories of minor compounds, among which polar phenols, oleuropein, and ligstroside derivatives, almost monopolized the interest of scientists the last thirty years for their health attributes [3].

Currently, three are the authorized health claims that can be used for commercial reasons on the VOO labels according to EC Regulation No 1924/2006 (article 13, paragraph 1) [4]. These claims are described in the EU Regulation No 432/2012 [5] that established a list of permitted health claims made on foods, ‘other than those referring to the reduction of disease risk and to children’s development and health’. Two of them are more generic in concept and refer to the nutrients oleic acid (C18:1) and vitamin E. In particular, the first refers to the practice of replacing saturated fats with unsaturated fats, such as oleic acid that is found in abundance in olive oil to ‘maintain normal blood cholesterol levels’. This health claim can be used for food in which unsaturated fatty acids represent at least 70% of total fatty acid content and provide more than 20% of the energy of the product. The second, which is also applicable to olive oil, refers to vitamin E and its contribution to the ‘protection of cells from oxidative stress’. This health claim can be used for food that is a source of vitamin E (at least 15% of 12 mg, which is the daily reference intake value for vitamin E supplied by 100 g of the product). The third health claim is dedicated to the ‘olive oil polyphenols’ and their contribution to the ‘protection of blood lipids from oxidative stress’. The use of this claim presupposes that the consumption of 20 g of olive oil provides at least 5 mg of hydroxytyrosol (Htyr) and its derivatives (e.g., oleuropein derivatives and tyrosol (Tyr). For a health claim to be used on the label of a product there must be proof of its trustworthiness. Reliable evidence can be obtained through analytical data. Official or validated protocols accepted by regulatory authorities (e.g., EU, FDA) or recommended by relevant organizations (e.g., IUPAC, AOAC, IOC (International Olive Council) are usually applied for such a purpose. Commonly, the determination of fatty acids is carried out using an EEC official method. Whereas, tocopherol analysis is accomplished using validated IUPAC or ISO protocols with or without slight modifications. There is no method widely accepted or fully validated for the determination of olive oil phenolic compounds for the implementation of the health claim. There has been significant effort taken in order to reach a consensus among interested parties for a harmonized and standardized protocol that can be recommended by the IOC or adopted by the EU [6]. This particular health claim can be implemented by interested parties if this is achieved.

VOO is a natural product, which is produced using olives from different cultivars, grown under different pedoclimatic and agronomic conditions and maturity stage at harvesting. These parameters qualitatively and quantitatively affect VOO’s composition, in terms of major and minor constituents. Extraction means and conditions (two or three-phase system, temperature, water addition, oxygen control) can further influence the content and forms of constituents present in the end product [7]. Over the past several decades, there has been substantial effort by the olive oil scientific community to systematically study the chemical composition of VOOs obtained from major and minor cultivars, either to establish official ranges for authentication purposes or to support trade names such as geographical indications and monovarietal origin. These studies are now expanded to cover the need to support health claims, too [8].

The literature for the above-mentioned health claim related compounds, present in Greek VOOs, is still scarce and partially updated or reviewed even for the cv. Koroneiki, which is the most important autochthonous cultivar for oil production and mainly thrives in South Peloponnese and Crete [9]. Chondrolia Chalkidikis and Chalkidiki cultivars that are cultivated almost exclusively in the regional unit of Chalkidiki (Map S1) are distinguished internationally as a source for high quality green table olives production. Only recently the interest of local producers and authorities is also turned to the production of high quality VOO. Registration of Agoureleo Chalkidikis [10] as a protected denomination of origin (PDO) product is an outcome of efforts to upgrade the production of VOO from these cultivars and support their sustainability in the region. Still, little is known on the potential of these cultivars regarding the above-mentioned health claims, the use of which may boost further consumer interest and market share for VOOs from cv. Chondrolia Chalkidikis and Chalkidiki.

The present work aims at evaluating the nutritional profile of the VOO from cv. Chondrolia Chalkidikis and Chalkidiki to bear health claims. Fatty acid, tocopherol, and polar phenol composition and content were examined for VOOs produced at industrial scale from different olive mills of Chalkidiki regional unit and also for VOOs obtained at laboratory scale from drupes of different maturity stages for four consecutive harvesting years. To our knowledge, this is the first systematic collection of data to be published for Chondrolia Chalkidikis and Chalkidiki VOO.

## 2. Materials and Methods

### 2.1. Chemicals and Other Materials

For HPLC analysis methanol (HPLC, 99.9%) and hexane (HPLC 95%) were purchased from Merck (Darmstadt, Germany). 2-Propanol (Chromasolv) was from Riedel de Haën (Seelze, Germany). Acetonitrile (HPLC, 99.9%), acetone (HPLC, 99.8%), and water (HPLC grade) were obtained from ChemLab (Zeldegem, Belgium). Phosphoric acid (≥85%) and sulphuric acid (95–98%) were supplied by Sigma-Aldrich (Steinheim, Germany). Sodium carbonate (anhydrous) and Folin-Ciocalteu (F-C) reagent were obtained from ChemLab. Other solvents and reagents were of the appropriate grade from various suppliers. Htyr (≥98%) was purchased from Extrasynthèse (Genay, France) and Tyr (≥98%) from AlfaAesar GmdH & Co KG, (Karlsruhe, Germany). Caffeic acid (CA) (98%) was from Sigma–Aldrich and α-Τ (>96%) were from Fluka Chemie GmbH (Buchs, Switzerland). Polyvinylidene fluoride (PVDF) membrane filters (0.22 µm) and cellulose acetate membrane filters (0.45 µm) were from Schleicher & Schuell, (Dassel, Germany).

### 2.2. Analytical Instrumentation

A UV-visible spectrophometer (Model UV-1601, Shimadzu Co., Tokyo, Japan) was used for the determination of total polar phenol (TPP) content, total chlorophyll (TChl) content, and the estimation of spectrometric indices at 232 and 270 nm. Data were processed using the UV Probe software (Version 2.3, Shimadzu Co.).

Fatty acid methyl ester analysis was carried out on an Agilent 6890 gas chromatograph (GC, Agilent Technologies, Palo Alto, CA, USA) equipped with a flame ionization detector (FID) (Agilent Technologies) and a split/splitless injector.

Determination of α-Τ was performed on an HPLC system equipped with a P4000 Finnigan MAT pump (Thermo Separation Products Inc., San Jose, CA, USA), a SCM1000 vacuum membrane degasser, a Midas autosampler (Spark, Emmen, The Netherlands), and a UV 6000 LP diode array detector (DAD; Thermo Separation Products Inc.) connected in series with an SSI 502 fluorescence detector (FLD; Scientific Systems Inc., State College, PA, USA). The Chrom Quest software (version 3.0, Thermo Separation Products) was used for HPLC data acquisition and analysis.

Determination of the total Htyr and Tyr content was carried out on a Shimadzu Nexera X2 UHPLC System, (Shimadzu Co.) equipped with a LC-30AD pump, SIL-30AC autosampler (50 µL loop), a CTO-20AC column oven, and a UV-visible diode array SPD-M30A detector (temperature-controlled semi-micro flow cell of 2.5 µL). The Lab Solution ver. 5.86 software (Shimadzu Co.) was used for UHPLC data acquisition and processing.

A portable spectrophotometer (MiniScan, Hunter Lab, Murnau, Germany) was used for olive oil color assessment. Other equipment used was an IKA MS3 digital vortex (IKA, Staufenim Breisgau, Germany), an ultrasound bath Elmasonic S 30 (H), (Elma Schmidbauer GmbH, Singen, Germany) [37 kHz; 80 W; Unit outer dimensions W/D/H, 137/100/151, mm/mm/mm, Capacity: 2.75 L], a HBA 200 benchtop centrifuge (Hettich Instruments LP, Tuttlingen, Germany) and a WB 3015 water bath (Bioline Scientific, Athens, Greece).

### 2.3. Sampling Design

#### 2.3.1. VOOs Produced at Industrial Scale

Sampling from fifteen olive mills of the regional unit of Chalkidiki, to cover the majority of the cv. Chondrolia Chalkidikis and Chalkidiki VOO production was conducted in the year 2016/17 (November–December 2016). The sampling zone is shown in Map S1. Table S1 shows relevant metadata for the olive mills. Samples were stored in dark bottles without headspace in the dark at room temperature (average temperature 17 and 26 °C for September-February and March-August, respectively) for eighteen (18) months.

#### 2.3.2. VOOs Produced at Laboratory Scale

In the next four consecutive harvest years (2017/18, 2018/19, 2019/20, 2020/21) VOOs were extracted under cold conditions from healthy olives cv. Chondrolia Chalkidikis and Chalkidiki harvested at different maturity stages using an Abencor laboratory olive mill (MC2, Ingenierías y Sistemas, Seville, Spain). The olives were harvested from 3 different olive groves (OG) in the area of N. Triglia (Chalkidiki, Greece). The OG1 [Location Coordinates, (Greek Coordinate System EGSA87/EPSG: 2100): x = 431,298.495, y = 4,461,830.341] had the following characteristics: fifty (50) trees aged about 7 years; distance between trees 6.5 × 7 m; clay soil; altitude 150 m; weekly drip irrigation with approx. 200 L of water per tree from 20 June to 15 September. OG2 and OG3 olive groves [Location Coordinates, (EGSA87/EPSG: 2100): x = 431,360.306, y = 4,461,770.941 and x = 431,445.678, y = 4,461,792.891 respectively] had the following characteristics: forty-five (45) trees about 12 years old, the distance between trees 6.5 × 6.5 m, red soil, altitude 150 m, weekly drip irrigation with about 200 L of water per tree from 20 June to 15 September. The sampling protocol was according to the guide COI/OH/Doc. No. 1 [11]. In each sample, 1.5–2 kg olives were hand-harvested from ten olive trees with comparable crop load and a similar maturity index in each OG. The olive trees were from the center of the OG; olives were picked from 3 levels (low, medium, high) around the tree. Sampling took place in the morning between 09:00–11:00 a.m. from early September to mid-November.

After harvesting, the maturity index (MI) was determined according to the procedure described by the IOC [11] based on the evaluation of olive skin and pulp color. VOO was extracted within 24 h. An amount of 1 kg of olives was washed and crushed with a hammer mill. The resulting olive paste was kneaded at a temperature of about 25 °C for 30 min and then centrifuged at 3500 rpm for 1 min. The extracted oil was collected in a calibrated volumetric cylinder to determine the extracted oil percentage by applying the Equation (1):

(1) $$\mathrm{Oil}\;\mathrm{percentage}\;\left(\%\right)=(\frac{\mathrm{mL}\;\mathrm{of}\;\mathrm{obtained}\;\mathrm{oil}\;\times \;0.915}{\mathrm{weight}\;\mathrm{of}\;\mathrm{the}\;\mathrm{paste}})\;\times \;100$$

After extraction, the color of the VOO was evaluated with a portable spectrophotometer and expressed as chromatic coordinates L*, a*, b*, adopted by the CIE Lab system. The average of triplicate values was taken for each sample. Relevant parameters, chroma value (C*) and hue angle value (h*), were calculated. Additionally, the total chlorophyll (TChl) content was also estimated according to an IUPAC standardized method proposed by Pokorny et al. [12]. Accordingly, the absorbance of the homogenized and filtered oil sample at wavelengths, 630, 670, and 710 nm was measured against air. Then, Equation (2) was applied. The result was expressed as pheophytin a, mg/kg. Samples were measured in triplicate:

(2) $$\mathrm{Pheo}\;a,\left(\mathrm{mg}/\mathrm{kg}\right)=345.3\;\times \;\frac{\left(A_{670}-\frac{A_{630}+A_{710}}{2}\right)}{L}$$

where A_λ_ is the absorbance of the oil at the respective wavelength and L the cell thickness (mm).

VOO samples (100 mL) were transferred in dark vials, which were then stored at −18 °C until further analysis.

### 2.4. Determination of Official Quality Indices

Free acidity (% oleic acid), peroxide value (PV) (meq O_2_/kg oil), and UV spectrophotometric indices at 232 and 270 nm (expressed as K_232_ and K_270_ values) were determined according to the methods described in the EEC Regulation No. 2568/91 [13] and its amendments. The repeatability of each method was checked (CV% = 4.8, 4.1, 4.9, and 5.2 for acidity, peroxide value, and K_232_, and K_272_, respectively, *n* = 5) and all parameters were then determined in duplicate for each sample.

### 2.5. Determination of Fatty Acid (FA) Composition (% Fatty Acid Methyl Esters, FAMEs)

The fatty acid composition was determined by GC as fatty acid methyl esters (FAMEs). FAMEs were prepared by transesterification with a methanolic solution of potassium hydroxide at room temperature according to Regulation (EEC) No. 2568/91 and its amendments. A capillary, TR-FAME column (60 m × 250 μm i.d., 0.25 μm) (ThermoScientific, Bellefonte, PA, USA) was used. The separation conditions were as follows: Carrier gas: helium (1.1 mL/min), the injector and detector temperature were set at 240 °C and the injection volume was 2 μL (split ratio 50:1). The temperature was programmed at 100 °C for 5 min, raised from 100 to 240 °C within 15 min, and held constant at 240 °C for 40 min. Identification of FAMEs was based on their retention times to the commercial standard FAME mixture (Sigma Chemical Co., St. Louis, MO, USA) in line with the standardized reference method. The percentages of the individual FAMEs were calculated from the total area of the peaks recorded on the chromatogram. Samples were analyzed in duplicate. Before analysis, the repeatability of the method was checked (CV% = 1.2, 1.5, 4.4, 0.4 and 1.2 for palmitic (C16:0), palmitoleic (C16:1), stearic (C18:0), oleic (C18:1), linoleic (C18:2) acids, respectively, *n* = 5).

### 2.6. Determination of α-T Content

Determination of α-T was performed on a LiChrospher-Si column (250 × 4 mm i.d., 5 mm) (MZ Analyzentechnik, Mainz, Germany) according to Psomiadou and Tsimidou [14]. The elution system was *n*-hexane/2-propanol (99:1 v/v) (A) and 2-propanol (B). The gradient composition for A was: 100% (10 min); 100–95% (10–14 min); 95% (14–20 min); 95–100% (20–24 min); 100% (24–30 min), the flow rate was 1.2 mL/min and the injection volume was 20 mL. The α-T standard (7.5–80 mg/L) and sample solutions (8% w/v) were prepared into the elution solvent and filtrated through a 0.45 μm membrane filter before injection. Quantification was carried out using an α-T calibration curve and fluorescence detection (λexc/λem = 294/330 nm). Samples were analyzed in duplicate (CV% = 4.2, *n* = 5).

### 2.7. Colorimetric Estimation of TPP Content

TPP content was determined colorimetrically using the Folin-Ciocalteu reagent according to the procedure proposed by Nenadis et al. [15]. The results were expressed as mg CA/kg by means of a calibration curve (50–500 mg/L). The determination for each extract was performed in triplicate.

### 2.8. UHPLC Determination of the Total Htyr and Tyr Content

For the determination of the total Htyr and Tyr content, an in-house validated UHPLC protocol proposed by Tsimidou et al. [16] was adopted. The polar fraction (PF) was prepared as described in the IOC protocol (COI/T.20/Doc No 29) [17] without the addition of an internal standard. For the acidic hydrolysis, an aliquot (200 μL) from the PF is mixed with 200 μL of a 1 M H_2_SO_4_ solution, and the mixture is incubated in a water bath at 80 °C for 2 h. The isolated PF and its hydrolysate were filtered through 0.22 µm PVDF membrane before injected onto the chromatograph. The chromatographic analysis was carried out on a 75 × 2.0 mm, 1.6 µm Shim-pack XR-ODS III Shimadzu column (Shimadzu Co.). The injection volume for PF or its hydrolysate was 3 µL. Htyr and Tyr before (Htyr_free_, Tyr_free_) and after hydrolysis (Htyr_hydrolysate_, Tyr_hydrolysate_) were quantified at 280 nm using the respective external calibration curves (0.5–100 mg/L). The total amount of Htyr and Tyr is calculated by applying Equation (3):

Total Htyr and Tyr, (mg/20 g) = [Htyr_free_] + [Tyr_free_] + 2.2 × [Htyr_hydrolysate_ − Htyr_free_] +2.5 × [Tyr_hydrolysate_ − Tyr_free_] (3)

## 3. Results and Discussion

### 3.1. VOO cv. Chondrolia Chalkidikis/Chalkidiki. VOOs Produced at Industrial Scale

#### 3.1.1. Generic Authorized Health Claims for Oleic Acid and Vitamin E

Quality characteristics were monitored just after delivery of samples (time zero) and after 6, 12, and 18 months of storage (Table S2). At time zero four of them were found not to comply with the official requirements for the extra virgin olive oil (EVOO) category and were not further analyzed. The eleven EVOOs showed rather low initial values regarding free acidity, peroxide value, and UV absorbances at 232 and 270 nm. At the end of storage, three samples exceeded the upper limit for at least one of these quality criteria. An analysis of the fatty acid composition (%) indicated that oleic acid percentage (initial mean value 74.5 ± 0.8) covered the official requirements for the respective health claim and its % content remained at similar levels throughout the storage period of 18 months (Table 1). Nevertheless, the mean initial value was found to fulfill marginally the limit set for the PDO product (≥75.0%). This information is an interesting finding that has to be considered by the interested parties in a future revision of the relevant dossier. Similar were the observations for the ratios C18:1/C18:2 and MUFA/PUFA (mean value 9.9 ± 1.4 and 9.2 ± 1.2, respectively) while the corresponding ranges reported for the PDO product seem to tend to much higher values (9.7–16.9, C18:1/C18:2; 9.1–15.7, MUFA/PUFA, respectively) (Table 1). Our finding has to be taken into consideration in a future revision of the technical information for the PDO Agoureleo Chalkidikis. The % composition of fatty acids at all points of testing over the 18-month storage is given in Table S3. In relation to the saturated fatty acids (SFA), C16:0 and C18:0 content ranged from 12.1–14.4% (mean value 13.3 ± 0.7), and from 1.8–2.3% (mean value 2.0 ± 0.2), respectively, while C20:0, and C22:0 were found in very low amounts (mean value 0.4 ± 0.0, and 0.1 ± 0.0, respectively). Finally, PUFA composition (C18:2, 6.5–10%/mean value 8.0 ± 1.3; C18:3 < 1.0%) indicated that the oil from the drupes of the two autochthonous cultivars could be affected by storage negatively unless this is kept under controlled temperature environment in the dark. The latter view is supported by the slow increase in the peroxide values and absorbance indices upon storage of the 11 extra virgin olive oils at ~21 °C mean temperature for 18 months, which is the lengthiest best before date permitted for this high quality edible oil (Table S2).

Information in literature for the fatty acid composition of this monovarietal VOO is limited. Kosma et al. [18] analyzed 21 VOOs cv. ‘Chondrolia Chalkidikis’ from the regional unit of Chalkidiki and Preveza (Epirus Greece) and reported similar mean values for C18:1 (73.5 ± 2.8), C18:1/C18:2 (8.2 ± 1.9) and MUFA/PUFA (7.7 ± 1.7) irrespective the location of the olive groves. The percentages found for the rest of the fatty acids are comparable. In terms of the fatty acid composition of VOOs from Koroneiki cv., the major Greek cultivar for oil production, it seems that ‘Chalkidiki’ cultivar yields oil lower in oleic acid and higher in linoleic acid content (Koroneiki cv VOO: C18:1 > 75% and C18:2 < 7.5%) [19,20,21]. This finding is in line with the general view for oils obtained from southern regions of the country [22].

The major tocopherol present in the VOOs obtained from the olive mills was α-T that accounted for 90–92% of the total tocopherol content. The homologues beta-tocopherol (β -Τ) and gamma-tocopherol (γ-T) were present at much lower and quite similar percentage ranges (4.7–5.3% and 3.7–4.7%, respectively). The initial absolute values of α-T and those evolved over the storage period are shown in Table 2.

The mean α-T content of the examined EVOOs was 202 mg/kg (range 166–263 mg/kg), which fell in the middle of the range reported for Greek EVOOs (98–370 mg/kg) [23], a fact that, looking at the metadata available, is at least partially explained by the implementation of good manufacturing practices in the olive mills of the region. To this point, it should be stressed that application of ‘cold’ technology (first cold pressing or cold extraction) and generally low malaxation temperatures do not favor tocopherol transfer from olive tissues [24]. The mean loss after eighteen months of storage was found to be ~27%, of similar magnitude to the one found (<20%) by Psomiadou and Tsimidou [25] after twenty-four months of storage in the dark at room temperature and by Ghanbari et al. [26] (~20%) after twelve months under similar storage conditions. Even so, the use of the respective health claim on the label could be valid throughout the storage period. Specifically, οn month 18th, the α-T content covered 18–30% of the daily reference intake for vitamin E (12 mg) [27] for a daily consumption dose of 20 g of VOO (2.2–3.5 mg/20 g).

Findings so far suggested that ΕVOO cv. Chondrolia Chalkidikis and Chalkidiki can bear safely the two generic health claims throughout the shelf life of the product. This optional labeling is of importance for consumer education, who are not fully aware of the compounds that are responsible for olive oil benefits and has not been exploited so far for commercial reasons [28].

#### 3.1.2. Dedicated Authorized Health Claim for ‘Polyphenols’

The mean initial TPP content observed for the EVOOs was 259 ± 79 mg/kg ranging from 141 to 445 mg/kg. This was the first indication of differences in agronomic and industrial practices applied in the Chalkidiki regional unit. These practices are expected to influence more the initial polar phenol content in comparison with what is expected for the lipophilic antioxidants [7]. The majority of the samples (8 out of 11) showed a total Htyr and Tyr content above the limit of 5 mg per 20 g oil and, thus, could bear the health claim on their label at the time of bottling (Table 3). Interestingly, the eight oils that fulfilled the requirement for the health claim maintained the necessary quantity of phenols throughout the 18 months of storage. (Table 3). This finding is very encouraging for those interested in promoting the PDO Agoureleo Chalkidikis by using on the label the health claim on ‘olive polyphenols’.

So far, the results of the experimental design indicate that the use of the three health claims in the marketing of EVOO from these two cultivars can be safely used by the local industry, distributors, and exporters.

### 3.2. VOOs Produced at Laboratory Scale

To better understand the potential of the two cultivars in contributing to the production of this VOO that can bear concomitantly three health claims, we carried out a study for the influence of MI on the content of oleic acid, α-T, and total Htyr and Tyr content. The study was expanded on different dates for four consecutive harvesting years and the oils were extracted at laboratory scale for obvious reasons. The date on which the producers harvested olives for oil production at industrial scale based on their experience for ‘technological optimum’ stage, oil was extracted from the same batch of olives at laboratory scale for comparison.

#### 3.2.1. Evolution of Olive MI, Oil Yield, and VOO Quality Parameters

The evolution of olive maturity was evaluated using two criteria, MI and % oil yield values (Table 4). The maturity stage except for oil yield affects quality parameters of the oil obtained and values for such parameters (mandatory or not) were also included in the same table. The first harvest date coincided with the harvesting onset under the council Regulation (EU) No 510/2006 for the PDO ‘Agoureleo Chalkidikis’ [29]. The respective MI values were found to be ~0.9 for all 4 years. Until the end of October (29/10) MI did not exceed values of 3.8. During the four-year research, the fruit of the examined cultivars tended to remain green irrespective MI value; the maximum MI value ranged between 3.5–4 until the end of the examined harvest period (16/11).

A similar ‘maturity evolution profile’ has been reported for the Frantoio cultivar for the same harvest period (16/9–16/11) with maximum MI values 2.2–3.7 and 3 consecutive harvest years (1996/97, 1997/98, and 1998/99). The cultivar has been characterized by Beltrán et al. [30] as a ‘late maturing’ cultivar, with the meaning of a drupe that ripens slowly. Maturation progress was slower in the year 2018/19 and faster in 2019/20 that was in line with meteorological evidence. According to the data obtained from the Hellenic National Meteorological Service [31], the average temperature recorded in the examined area was 16.8 °C during the 2017/18 harvest period and higher by 1.2, 1.9, and 1.6 °C the next three sampling periods (2018/19, 2019/20, and 2010/21), respectively. Taking into account that the agronomic practices (e.g., irrigation) applied to the three olive groves were the same in the four years of the study as they are centrally controlled by the municipality and slight temperature variance observed, fruit load varied, the lowest reported by the producer in the fourth year of the study.

An increase in the percentage oil yield was observed up to the end of October while then until the end of the harvest period decreased (Table 4). The percent yield values varied from year to year (6.9–22.1%, 8.0–14.4%, and 11.0–14.0% for 2017/18, 2019/20, and 2020/21, respectively. In the year 2018/19 a shorter harvest period was evidenced (ended on 22/10) and the % oil yield was the lowest one (6.6–11.7%). These values confirm the yield range (14–20%) reported for the cultivars Chondrolia Chalkidikis and Chalkidiki, which are characterized as cultivars of a medium oil yield [29].

Total chlorophyll content fluctuated from year to year and was reduced significantly upon ripening. Nevertheless, this dependence was not reflected clearly on the values for the color parameters C* and h*, which were also assessed just after the laboratory extraction process (Table 4). The calculated value of chroma (C*), which expresses the intensity or purity of the color decreased with an increase in MI values; ranging from 104–65, 81–74, 67–51, and 88–65 during the periods 2017/18, 2018/19, 2019/20, and 2020/21, respectively. The color of VOO at the end of the harvest period was more yellow to gold, as evidenced by the reduction in the value of hue angle (h*) (from 98° to 90°, from 91° to 87°, from 99° to 89°, and from 92° to 88° during the harvest years 2017/18, 2018/19, 2019/20 and 2020/21, respectively). The prevailing of yellowish hues was in line with the progressive reduction in total chlorophyll content of the oil samples. Τhe total chlorophyll content reached a maximum in VOOs from olives at MI 0.9 (50.2, 38.9, 25.9, and 52.1 mg pheo a/kg oil for the harvest year 2017/18, 2018/19, 2019/20, and 2020/21 respectively. The percentage decrease at the end of the monitoring period was 93% (2017/18), 55% (2018/19), 97% (2019/20), and 85% (2020/21) (Table 4). Similar percentage reductions (97.4 and 82.4%) have been reported for cultivars Picual, and Hojiblanca, respectively, at the end of maturation (MI 5–5.5) [32].

The evolution of the official quality criteria values was examined to check whether the harvest date could lead to oils non-compliant with requirements for the best commercial quality category ‘extra’. The results are presented in Table 4. The value of acidity slightly increased, as maturation progressed in all harvest years but did not exceed the limit of 0.8% established for the ‘extra’ category. This trend may be due to an increase in enzymatic activity, especially by lipolytic enzymes in the olive fruit during maturation or in the olive paste during malaxation. The degree of activity of enzymes depends on MI, temperature, or humidity, and does not require prior insect infestation or fruit damage [33,34]. PV of VOOs cv Chondrolia Chalkidikis and Chalkidiki was lower in the late harvest dates, which may be due to the reduction in the enzymatic activity of lipoxygenase in line to what has been reported for oils from other cultivars such as Koroneiki, Chemlali, Chétoui, Picual, and Hojiblanca [32,33,34,35,36]. However, at intermediate MIs an increase in PV was observed and then a decrease until the end of the harvest period (except 2020/21, which remained almost constant). Salvador et al. [37] also observed a higher PV value in VOO cv. Cornicabra obtained from olives at MI 4.1 vs that obtained from olives at MI 4.4. The absorbance indices K_232_ and K_270_ did not show a clear dependence on maturation. All the VOOs produced and analyzed showed low initial values for the official quality indices and all of them belonged to the ‘extra’ category.

The samples Mill17_29/10, Mill18_22/10, Mill19_16/11, and Mill20_29/10 are of particular interest to the study. These VOOs were obtained from olives that were harvested on the date set by the producer as suitable for extraction in each of the harvest years (29/10 in 2017/18, 22/10 in 2018/19, 16/11 in 2019/20, and 29/10 in 2020/21). These samples were extracted by a centrifugal two-phase system in an olive mill of the studied area. Values for the official quality parameters (acidity: 0.23, 0.73, 0.53 and 0.62%, PV: 8.1, 13.3, 8.2 and 14.0 meq O_2_/kg, K_270_: 1.47, 1.86, 1.54 and 1.7, K_232_: 0.14, 0.09, 0.11 and 0.13) showed that these industrial samples were also classified as EVOOs though the corresponding values were higher from those for the oils obtained at laboratory scale, indicating that the potential of the cultivar may be affected negatively by the manufacturing practices at the olive mill.

#### 3.2.2. Evolution of Monounsaturated Fatty Acid Content

Changes in the fatty acid percentages during maturation showed similar trends in all harvest years (Table 5). On the first harvest date, palmitic acid content was the highest with a mean value of 13.4%, then decreased slightly, and reached a plateau of a mean value of 12.4 at higher MI values. A reduction in palmitic acid level has been reported for several olive cultivars during maturation [34,36,38,39,40] and has been attributed to a dilution effect rather than in biosynthetic reasons [32]. Changes in palmitic acid levels slightly fluctuated from year to year. Specifically, the palmitic acid level remained almost constant in VOOs produced in 2019/20 and 2020/21 up to a MI of 3.6–3.8. The stearic acid presented the maximum level of 2.7 % at a MI above 3.5. The maximum level of 4.4 % for stearic acid when the MI reached 5.5 was also reported for VOO cv. Souri [40] indicating its accumulation during maturation. Oleic acid exhibited a decreasing trend from a mean value of 74.3% at the beginning of the harvest period to 70.5% at MI between 3.7 and 4.0. This decrease was not continuous in 2019/20 and 2020/21, in which a sharp increase was observed at MI 3.8, and 3.3, respectively. Linoleic acid showed an opposite trend as the highest percentages were observed at higher MIs. As a result, a substantial decrease in oleic/linoleic acid and MUFA/PUFA ratio was observed during maturation, which can be attributed to the transformation of oleic acid into linoleic acid by the enzyme oleate desaturase, which is active during triacylglycerol biosynthesis [32], as well as the high field temperatures [41]. Lower values of MUFA/PUFA ratio have been reported for VOOs produced in warm seasons and areas [41]. During summer, daily temperatures did no exceed 33 °C for all harvest years; the lowest mean temperature (27 °C) was recorded in 2020–2021. The MUFA/PUFA ratio in the VOOs cv. Chondrolia Chalkidikis and Chalkidiki at MI 3.7–4.0 presented half value (5.5, *n* = 3) to the corresponding one (10.4) for a VOO cv. Koroneiki from olive fruit of the same MI [42]. The latter did not necessarily indicate lower stability of VOOs cv. Chondrolia Chalkidikis and Chalkidiki as its oxidative status is also strongly affected by the concentration in natural antioxidants. The fatty acid composition of the samples from the olive mill showed intermediate values between those observed for samples extracted at laboratory scale (Table 4). The VOOs stood out for their high content of oleic acid, which permits the use of the respective health claim.

#### 3.2.3. Evolution of α-T Content

The effect of the maturity process was evident in the content of α-T, which decreased with the maturity progress in all harvest years as presented in Table 6. A higher content was observed in September, which did not particularly vary between harvest years (219, 236, 170, and 176 mg/kg in 2017/18, 2018/19, 2019/20, and 2020/21, respectively). Until 15/10 there was a decrease of ~20% in the α-T content (except in 2020/21 when there was an increase in α-T content) and then remained practically constant during the rest of the harvest period. This trend has also been reported for other Greek and Portuguese cultivars [42,43]. The percentage of β-Τ, which was around 5%, remained almost constant, while γ-T with a similar percentage range presented a slight decrease (~1%) during maturation. The α-T content for the VOOs produced at industrial scale was also intermediate between those found for VOOs extracted using the Abencor system (Table 6), which verifies the safe use of the health claim for Vitamin E on the label of this extra virgin olive oil.

#### 3.2.4. Evolution of the TPP and the Total Htyr and Tyr Content

Changes in the content of TPP and the total Htyr and Tyr with increasing MI value are presented in Table 7. The ΤPP content showed differences between harvest years with the highest being recorded in the year 2019/20 (370–916 mg/kg vs. 198–676, 218–515, and 371–463 mg/kg during the year 2017/18, 2018/19, and 2020/21, respectively). TPP content increased gradually until reached a maximum at MI values between 2.0 and 2.7 and then decreased until the end of the harvest period. This tendency may be because phenolic compounds gradually accumulate in the olive fruit similar to oil, reaching a maximum concentration just as the olive fruit begins to change color, and concomitantly the water content decreases (MI values between 1–2) [44]. However, in 2017/18 and 2020/21 the maximum content was attained earlier (MI ~1). The same trend was observed for the total Htyr and Tyr content with the maximum values being recorded in VOOs derived from olives with a range of MI 2.0–3.8. The total Htyr and Tyr content presented high values (8–24, 10–13, 12–21, and 11–14 mg/20 g oil in 2017/18, 2018/2019, 2019/20, and 2020/2,1 respectively) indicating that VOO cv. Chondrolia Chalkidikis and Chalkidiki exhibits a strong potential to bear the health claim until the end of the harvest period (16/11). The Htyr/Tyr ratio [45] for the examined cultivars did not present a specific trend during maturation and ranged from 0.31 to 0.97. All VOOs from the olive mill covered the official requirement for the respective health claim but the wide variation presented in their content indicated the need for good practices at industrial scale (Mill20_29/10 was found to fulfill marginally the limit set for the health claim).

## 4. Conclusions

The examination of VOOs from different olive mills of the Chalkidiki regional unit points out the need for controlled practices if the local industry wants to benefit from the optional health claim labeling for these products of cv Chondrolia Chalkidikis and Chalkidiki, irrespective of PDO or not. Most of the samples belonged to the extra category and remained so for up to 18 months of storage in the dark at room temperature. During storage, their nutritional profile met the requirements for the three authorized health claims (‘oleic acid’, ‘vitamin E’, and ‘olive oil polyphenols’). Finally, the examination of the stage of maturation of the cv. Chondrolia Chalkidikis and Chalkidiki olive drupes showed that these cultivars mature slowly. The best results in terms of content of oleic acid, α-Τ, and polar phenolic compounds were found for VOO with a maturity index of around 2. The results of these four consecutive year study can prove very useful to producers and the local olive industry in organizing the sector on a scientific basis.

## Figures and Tables

**Table 1:** %SFA, %UFA, C18:1 %FAME, C18:1/C18:2 and MUFA/PUFA ratio of VOOs cv. Chondrolia Chalkidikis and Chalkidiki from the main olive mills of the regional unit of Chalkidiki (2016/17).

Samples	Storage Time (months)	SFA	UFA	C18:1	C18:1/C18:2	MUFA/PUFA	Samples	Storage Time (months)	SFA	UFA	C18:1	C18:1/C18:2	MUFA/PUFA
1	0	15.5	84.5	74.8	9.7	9.2	7	0	16.0	84.0	74.5	10.4	9.7
	6	15.1	84.9	75.1	9.8	9.3		6	15.6	84.4	75.0	10.6	9.8
	12	15.1	84.9	75.1	9.9	9.3		12	15.6	84.4	75.0	10.6	9.9
	18	15.2	84.8	75.2	9.9	9.4		18	15.6	84.4	75.1	10.7	9.9
2	0	16.0	84.0	75.0	11.1	10.2	8	0	16.0	84.0	74.4	10.1	9.5
	6	15.4	84.6	75.0	11.4	10.5		6	15.0	85.0	75.4	10.2	9.6
	12	15.4	84.6	75.7	11.4	10.5		12	15.1	84.9	75.4	10.3	9.7
	18	15.3	84.7	75.8	11.6	10.7		18	15.1	84.9	75.4	10.4	9.7
3	0	16.0	84.0	75.1	10.8	10.0	9	0	14.8	85.2	74.9	8.9	8.6
	6	15.7	84.3	75.1	10.7	10.0		6	14.3	85.7	75.2	8.9	8.5
	12	15.5	84.5	75.1	11.0	10.2		12	14.3	85.7	75.1	8.9	8.5
	18	15.7	84.3	75.4	10.8	10.0		18	14.3	85.7	75.2	8.9	8.5
4	0	16.6	83.4	74.2	11.0	10.1	10	0	14.7	85.3	74.1	8.0	7.6
	6	16.1	83.9	74.9	11.1	10.3		6	14.3	85.7	74.2	7.8	7.5
	12	15.9	84.1	75.1	11.2	10.3		12	14.2	85.8	74.3	7.8	7.5
	18	15.7	84.3	75.3	11.2	10.3		18	14.4	85.6	74.2	7.9	7.5
5	0	15.6	84.4	72.3	7.2	6.8	11	0	16.2	83.8	75.1	11.5	10.7
	6	15.1	84.9	72.9	7.3	7.0		6	16.1	83.9	73.2	8.3	7.8
	12	15.1	84.9	73.0	7.4	7.0		12	15.0	85.0	76.4	11.9	11.1
	18	15.3	84.7	72.8	7.4	7.0		18	14.9	85.1	76.4	11.8	11.0
6	0	15.3	84.7	74.9	9.7	9.1							
	6	14.5	85.5	76.1	10.1	9.5							
	12	14.3	85.7	76.1	9.5	10.1							
	18	15.1	84.9	75.1	9.7	9.1							

SFA: saturated fatty acids; UFA: unsaturated fatty acids; C18:1: oleic acid; % FAME: % fatty acid methyl ester, C18:2: linoleic acid; MUFA: monounsaturated fatty acids; PUFA polyunsaturated fatty acids.

**Table 2:** α-Tocopherol (α-Τ) content (mg/kg oil) of VOO samples cv. Chondrolia Chalkidikis and Chalkidiki from the main olive mills of the regional unit of Chalkidiki during the 18-month storage in the dark at room temperature.

α-Τ mg/kg Oil									
Storage Time (months)									
Sample	0	6	12	18	Sample	0	6	12	18
1	189	189	154	149	7	205	205	185	140
2	235	223	185	173	8	204	202	167	160
3	224	217	176	164	9	147	144	114	110
4	263	234	188	177	10	168	151	120	114
5	174	162	127	118	11	189	188	182	133
6	166	160	134	139					

Mean values (*n* = 2).

**Table 3:** Total hydroxytyrosol (Htyr) and tyrosol (Tyr) content and total polar phenol (TPP) content of VOO samples cv. Chondrolia Chalkidikis and Chalkidiki from the main olive mills of the regional unit of Chalkidiki during the 18-month storage in the dark at room temperature.

Sample	TPP mg/kg *	Total Htyr+Tyr mg/20 g **				Sample	TPP mg/kg *	Total Htyr + Tyr mg/20 g **			
	Storage Time (months)										
	0	0	6	12	18		0	0	6	12	18
1	247	5	5	5	5	7	261	7	5	6	6
2	348	8	6	7	5	8	184	4	3	3	3
3	211	3	3	3	3	9	290	6	5	4	4
4	215	4	3	4	3	10	445	8	7	6	6
5	255	8	6	7	7	11	240	9	8	7	7
6	277	7	6	7	7						

* Mean values (*n* = 2); ** The total amount of Htyr and Tyr is calculated as the sum of the mean value of three replicates of total Htyr and mean value of three replicates of total Tyr; the sum is then rounded to the first integer.

**Table 4:** Effect of the harvest date on the maturity index (MI) of olives cv. ‘Chondrolia Chalkidikis’ and ‘Chalkidiki’, on the extracted oil yield and quality indices of the VOOs extracted from οlives cv. Chondrolia Chalkidikis and Chalkidiki at different stages of maturity for four consecutive harvest years (2017/18, 2018/19, 2019/20, 2020/21).

Harvest Year	Harvest Date	ΜΙ	% Oil Yield	C **	h **	TChl **	Acidity * (%oleic acid)	PV * (meqO_2_/kg)	K_232_ *	Κ_270_ *
VOOs produced at laboratory scale										
2017/18	15/9	0.9 ± 0.0	6.9	65.4 ± 0.5	98.3 ± 0.0	50.2 ± 0.1	0.22	6.3	1.83	0.13
	8/10	2.0 ± 0.4	15.5	102.5 ± 2.2	94.6 ± 0.1	35.2 ± 0.1	0.22	5.8	1.95	0.13
	15/10	2.7 ± 0.5	17.1	88.4 ± 0.5	90.8 ± 0.0	16.8 ± 0.1	0.23	3.3	1.43	0.09
	22/10	3.2 ± 0.4	19.8	92.5 ± 0.7	91.4 ± 0.1	15.9 ± 0.1	0.23	6.0	1.71	0.13
	29/10	3.3 ± 0.5	19.8	91.6 ± 0.9	91.2 ± 0.1	14.8 ± 0.0	0.23	3.7	1.57	0.14
	16/11	3.7 ± 0.4	15.2	96.2 ± 0.2	90.8 ± 0.0	3.1 ± 0.0	0.28	1.6	1.36	0.10
2018/19	15/9	0.9 ± 0.1	6.6	81.1 ± 4.0	91.4 ± 0.1	38.9 ± 0.1	0.34	8.5	1.56	0.09
	3/10	1.3 ± 0.2	10.8	71.9 ± 0.7	90.7 ± 0.1	32.8 ± 0.1	0.33	8.2	1.73	0.13
	8/10	1.7 ± 0.6	11.4	75.7 ± 4.4	90.5 ± 0.1	32.6 ± 0.1	0.32	8.6	1.63	0.12
	15/10	2.3 ± 0.7	11.6	77.2 ± 1.6	89.4 ± 0.0	23.2 ± 0.0	0.32	10.3	1.70	0.13
	22/10	2.4 ± 0.5	11.7	73.9 ± 7.9	89.4 ± 0.2	24.8 ± 0.0	0.34	6.1	1.56	0.09
2019/20	15/9	0.8 ± 0.0	8.0	50.6 ± 1.9	98.8 ± 0.3	25.9 ± 0.1	0.34	8.8	1.79	0.14
	3/10	1.4 ± 0.1	8.3	54.6 ± 0.7	94.6 ± 0.1	30.8 ± 0.0	0.34	8.7	1.55	0.12
	8/10	2.1 ± 0.1	9.7	62.9 ± 0.7	91.3 ± 0.1	24.7 ± 0.1	0.37	8.5	1.67	0.15
	15/10	3.1 ± 0.3	12.7	73.2 ± 0.5	89.5 ± 0.0	9.5 ± 0.0	0.34	8.3	1.81	0.16
	22/10	3.6 ± 0.2	13.2	62.8 ± 0.4	92.1 ± 0.1	4.2 ± 0.2	0.42	8.1	1.54	0.13
	29/10	3.8 ± 03	14.4	67.0 ± 1.9	89.2 ± 0.1	5.5 ± 0.0	0.42	6.4	1.63	0.13
	16/11	4.0 ± 0.2	8.3	49.1 ± 0.8	89.1 ± 0.0	0.74 ± 0.02	0.51	7.6	1.50	0.10
2020/21	15/9	1.0 ± 0.0	11.0	64.8 ± 5.2	92.0 ± 0.8	52.07 ± 0.78	0.28	9.2	1.66	0.12
	3/10	2.2 ± 0.5	14.2	67.4 ± 0.7	91.3 ± 0.2	36.99 ± 0.19	0.25	10.2	1.49	0.11
	22/10	3.3 ± 0.4	14.0	75.2 ± 1.5	88.2 ± 0.3	24.22 ± 0.02	0.31	10.6	1.62	0.10
	29/10	3.8 ± 0.3	14.0	87.5 ± 1.9	88.3 ± 0.4	7.83 ± 0.02	0.36	10.0	1.49	0.11
VOOs produced at industrial scale										
2017/18 Mill17_29/10	29/10	3.3 ± 0.5	n.a.	103.4 ± 06	90.2 ± 0.0	18.29 ± 0.09	0.23	8.1	1.47	0.14
2018/19 Mill18_22/10	22/10	2.4 ± 0.5	n.a	85 ± 3.4	86.8 ± 0.1	17.40 ± 0.01	0.73	13.4	1.77	0.11
2019/20 Mill19_16/11	16/11	4.1 ± 0.2	n.a	59.8 ± 0.6	86.8 ± 0.1	5.74 ± 0.04	0.53	8.2	1.54	0.11
2020/21 Mill20_29/10	29/10	3.8 ± 0.3	n.a	95.7 ± 0.2	84.8 ± 0.2	11.46 ± 0.22	0.62	14.0	1.70	0.13

* Mean values (*n* = 2); ** Mean value ± standard deviation (*n* = 3); n.a. = not available from the mill records; PV: peroxide value; C*: chroma value; h*: hue angle value; TChl: total chlorophyll content.

**Table 5:** Effect of the harvesting date on the % fatty acid composition of the produced VOOs for four consecutive harvest years (2017/18, 2018/19, 2019/20, 2020/21).

% Fatty Acid Methyl Ester Composition *										
Harvest Year	Harvest Date	C16:0	C16:1	C18:0	C18:1	C18:2	C18:3	C20:0	C18:1/C18:2	MUFA/PUFA
		VOOs produced at laboratory scale								
2017/18	15/9	13.4	1.3	1.7	74.9	7.0	0.7	0.4	10.7	9.9
	8/10	12.6‘	1.2	2.0	73.1	9.4	0.6	0.3	7.8	7.4
	15/10	12.9	1.2	2.0	71.8	10.4	0.7	0.3	6.9	6.6
	22/10	12.0	1.1	2.2	72.7	10.6	0.6	0.3	6.9	6.6
	29/10	11.9	1.0	3.1	71.7	10.8	0.6	0.3	6.6	6.4
	16/11	12.0	1.0	2.7	70.0	12.6	0.6	0.3	5.6	5.4
2018/19	15/9	13.2	1.2	1.8	74.6	7.3	0.8	0.4	10.2	9.5
	3/10	13.1	1.3	1.9	73.5	8.7	0.7	0.3	8.4	8.0
	8/10	12.2	1.0	2.0	74.4	8.4	0.7	0.5	8.9	8.4
	15/10	12.5	1.0	2.1	73.0	9.5	0.7	0.4	7.6	7.3
	22/10	12.9	1.1	2.0	72.8	9.4	0.7	0.4	7.7	7.3
2019/20	15/9	13.1	0.9	1.9	74.5	8.0	0.7	0.3	9.3	8.8
	3/10	13.1	1.0	1.8	73.4	9.1	0.6	0.4	8.1	7.7
	8/10	13.1	1.1	1.9	72.6	9.8	0.6	0.3	7.4	7.1
	15/10	13.1	1.0	2.1	71.1	11.0	0.7	0.5	6.5	6.2
	22/10	13.2	0.9	2.3	70.6	11.5	0.7	0.3	6.2	5.9
	29/10	12.5	0.8	2.0	74.8	9.6	0.6	0.3	7,8	7.4
	16/11	12.7	1.0	2.7	70.9	10.8	0.5	0.3	6.5	6.3
2020/21	15/9	13.7	1.0	1.8	73.3	8.6	0.7	0.3	8.6	8.1
	3/10	13.2	0.9	2.1	71.9	10.4	0.6	0.4	6,9	6.6
	22/10	12.5	0.8	1.6	77.1	6.1	0.7	0.4	12.6	11.4
	29/10	13.5	0.8	2.4	68.3	13.6	0.6	0.4	5.0	4.9
VOOs produced at industrial scale										
2017/18 Mill17_29/10	29/10	11.7	0.9	2.3	74.1	9.3	0.6	0.4	8.0	7.6
2018/19 Mill18_22/10	22/10	13.3	1.3	1.7	73.6	8.5	0.7	0.3	8.6	8.2
2019/20 Mill19_16/11	16/11	12.1	1.1	2.3	74.9	8.2	0.6	0.3	9.1	8.6
2020/21 Mill20_29/10	29/10	12.7	0.8	2.6	69.8	12.5	0.7	0.3	5.6	5.4

* Mean values (*n* = 2); fatty acids: palmitic, C16:0; palmitoleic, C16:1; stearic, C18:0; oleic, C18:1; linoleic, C18:2; linolenic, C18:3, arachidic, C20:0; MUFA: monounsaturated fatty acids; PUFA: polyunsaturated fatty acids.

**Table 6:** Variability in the α-Tocopherol (α-Τ) content of VOOs extracted from olives cv. Chondrolia Chalkidikis and Chalkidiki at different stages of maturity for four consecutive harvest years.

		α-Τ mg/kg		
Harvest Date	2017/18	2018/19	2019/20	2020/21
VOOs produced at laboratory scale				
15/9	219	236	170	176
3/10	-	203	190	177
8/10	197	210	185	-
15/10	173	195	135	192
22/10	171	199	135	-
29/10	179	-	132	153
16/11	177	-	122	-
VOOs produced at industrial scale				
22/10	-	217	-	-
29/10	215	-	-	185
16/11	-	-	119	-

**Table 7:** Variability in the total polar phenol (TPP), total hydroxytyrosol (Htyr) and tyrosol (Tyr) content, and ratio Htyr to Tyr of VOOs extracted from olives cv. Chondrolia Chalkidikis and Chalkidiki at different stages of maturity for four consecutive harvest years (2017/18, 2018/19, 2019/20, 2020/21).

Harvest Date	2017/18			2018/19			2019/20			2020/21		
	TPPC * mg/kg	Total Htyr + Tyr mg/20 g	Htyr/Tyr	TPPC * mg/kg	Total Htyr + Tyr mg/20 g	Htyr/ Tyr	TPPC * mg/kg	Total Htyr + Tyr mg/20 g	Htyr/ Tyr	TPPC * mg/kg	Total Htyr + Tyr mg/20 g	Htyr/ Tyr
VOOs produced at laboratory scale												
15/9	676 ± 49	24	0.44	372 ± 7	13	0.60	508 ± 11	12	0.70	463 ± 40	14	0.48
3/10	-	-		491 ± 10	20	0.66	819 ± 15	19	0.65	415 ± 9	12	0.54
8/10	673 ± 28	26	0.46	495 ± 26	17	0.82	916 ± 34	21	0.50	-	-	
15/10	311 ± 11	13	0.31	515 ± 8	9	0.54	881 ± 10	19	0.47	371 ± 2	12	0.58
22/10	437 ± 11	15	0.51	440 ± 20	10	0.48	697 ± 31	20	0.43	-	-	
29/10	273 ± 6	15	0.52	-	-		798 ± 58	19	0.97	430 ± 5	11	0.91
16/11	198 ± 9	8	0.97	-	-		794 ± 7	12	0.48	-	-	
VOOs produced at industrial scale												
22/10-	-	-		218 ± 4	5	0.45	-	-		-	-	
29/10	237 ± 12	13	0.38	-	-		-	-		271 ± 3	8	
16/11	-	-		-	-		370 ± 5	8	0.70	-	-	

* mean values ± standard deviation (*n* = 3).

## Data Availability

The data presented in this study are available on request from the corresponding author.

## Supplementary Materials

The following are available online, Map S1: Olive mills in Chalkidiki regional unit involved in the sampling design, Table S1: Location, extraction system, production capacity, and malaxation temperature of the olive mills involved in the sampling design, Table S2: Changes in the values of the official quality indices of VOO samples cv. Chondrolia Chalkidikis and Chalkidiki from the main olive mills of the regional unit of Chalkidiki during the 18-month storage in the dark at room temperature, Table S3: Changes in FA composition (%FAMEs) of VOOs cv. ‘Chondrolia Chalkidikis’ and ‘Chalkidiki’ from the main olive mills of the regional unit of Chalkidiki (2016/17) during the 18-month storage in the dark at room temperature.
