# Supplementary material for: The Potential of Virgin Olive Oil from cv. Chondrolia Chalkidikis and Chalkidiki (Greece) to Bear Health Claims according to the European Legislation
Source: Molecules. 2021 May 26;26(11):3184. doi: 10.3390/molecules26113184 (PMC8199220; doi:10.3390/molecules26113184)
Supplement: Supplementary file 1 [file molecules-26-03184-s001.zip › molecules-1217042-supplementary.pdf]

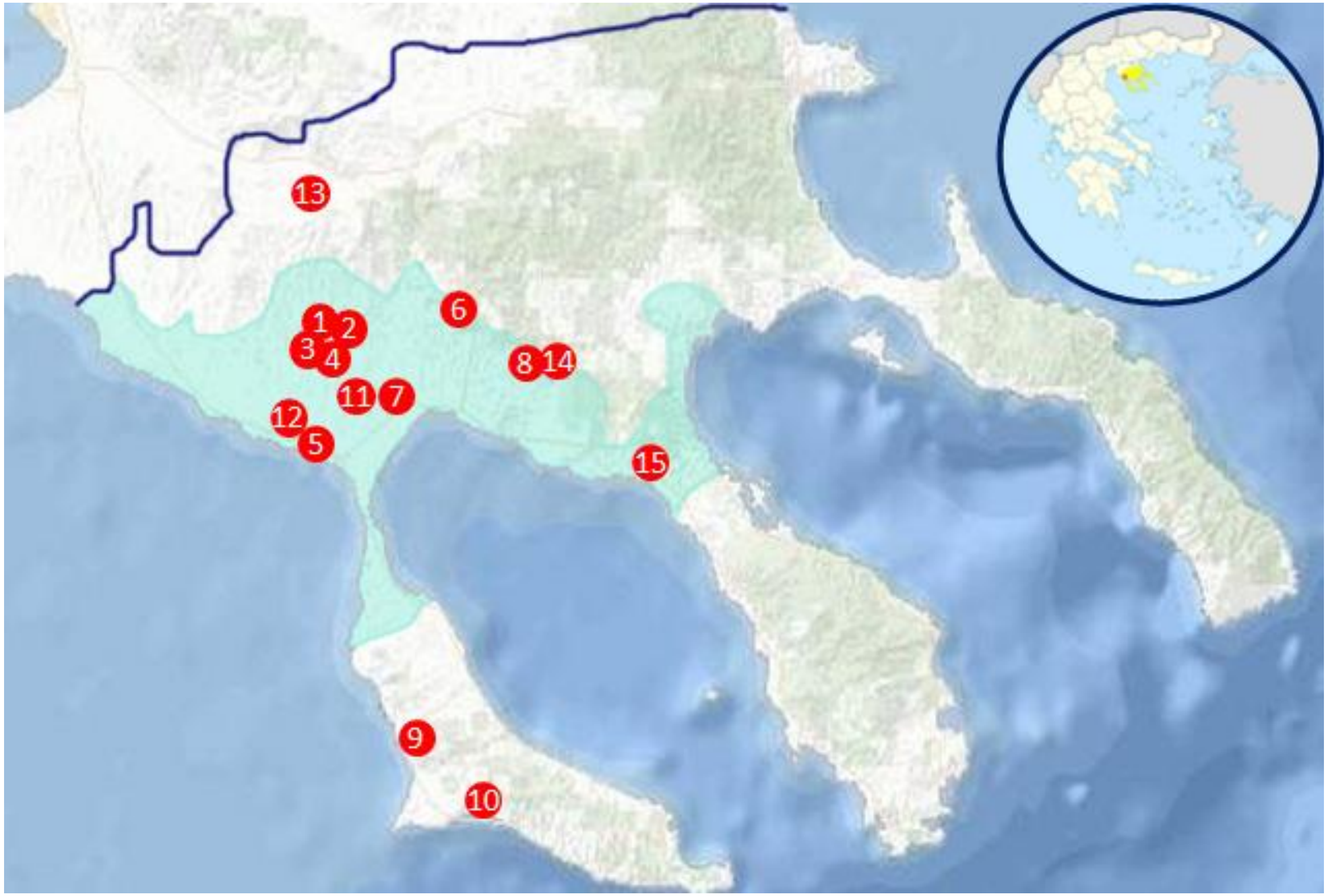

Map S1. Olive mills in Chalkidiki regional unit involved in the sampling design. The shaded light green color area reflects the highest olive growing activity in Chalkidiki

Table S1. Location, extraction system, production capacity and malaxation temperature of the olive mills involved in the sampling design.

Sample	Olive mill/location	Extraction system	Production capacity	Malaxation temperature	Sample	Olive mill/location	Extraction system	Production capacity	Malaxation temperature
1	Olive mill 1/Simandra	three-phase	4 t/h	25–30°C	9	Olive mill 9/Fourka	three-phase	8 t/h	29–32°C
2	Olive mill 2/Simandra	two-phase	4 t/h	22–23°C	10	Olive mill 10/Kalandra	three-phase	2 t/h	-
3	Olive mill 3/Portaria	three-phase	4 t/h	28–33°C	11	Olive mill 11/Olynthos	three-phase	3 t/h	~27°C
4	Olive mill 4/Portaria	two-phase	12 t/h	22–23°C	12	Olive mill 12/Dionisiou	three-phase	2.5t/h	25–28°C
5	Olive mill 5/N. Moudania	three-phase	2.5 t/h	~38°C	13	Olive mill 13/Galatista	three-phase	2.5t/h	30–32°C
6	Olive mill 6/Polygyros	three-phase	2,5 t/h	-*	14	Olive mill 14/Ormylia	three-phase	4 t/h	~38°C
7	Olive mill 7/Kalyves	three-phase	5 t/h	30°C	15	Olive mill 15/Nikiti	three-phase	3 t/h	28–30°C
8	Olive mill 8/Ormylia	three-phase	2 t/h	-					

*-: data not provided by the mill operator

Table S2. Changes in the values of the legal quality indices of VOO samples cv. Chondrolia Chalkidikis and Chalkidiki from the main olive mills of the regional unit of Chalkidiki during the 18-month storage in the dark at room temperature.

	Storage time (months)											
Sample	0	6	12	18	0	6	12	18	0	6	12	18
	Acidity* (% oleic acid) Upper limit: 0.8				PV* (meqO_2/kg) Upper limit: 20				K_{232}*/K_{270}* Upper limits: 2.5/0.25			
1	0.23	0.23	0.22	0.28	8.6	10.5	10.4	10.6	2.14/0.11	2.10/0.10	2.06/0.09	2.10/0.16
2	0.45	0.51	0.59	0.62	7.7	11.7	11.0	11.6	2.03/0.10	2.29/0.12	2.32/0.14	2.40/0.16
3	0.42	0.45	0.50	0.50	6.2	7.8	10.6	10.6	1.76/0.08	1.84/0.08	1.99/0.10	2.12/0.14
4	0.45	0.45	0.56	0.56	9.8	10.0	10.50	10.9	1.87/0.11	2.15/0.11	2.12/0.12	2.07/0.15
5	0.56	0.56	0.76	0.79	9.8	10.1	10.3	10.9	1.75/0.14	1.90/0.13	2.21/0.17	2.28/0.20
6	0.34	0.34	0.42	0.51	5.8	8.2	9.2	9.7	1.85/0.12	2.14/0.12	2.06/0.11	2.35/0.17
7	0.62	0.62	0.62	0.78	9.5	9.4	10.8	11.4	1.47/0.08	2.23/0.12	2.32/0.16	2.56/0.20
8	0.80	0.87	0.93	0.95	7.9	8.4	10.9	10.9	1.47/0.08	2.10/0.08	1.86/0.12	2.20/0.11
9	0.45	0.45	0.64	0.67	6.9	7.6	8.14	9.5	1.76/0.11	2.2/0.11	1.83/0.11	2.23/0.11
10	0.28	0.28	0.42	0.42	7.9	9.1	9.7	11.7	2.03/0.10	2.01/0.10	2.20/0.11	2.23/0.11
11	0.56	0.56	0.59	0.96	8.0	9.2	9.9	10.8	1.91/0.08	2.23/0.11	2.03/0.12	2.09/0.14

Mean values (n = 2); Values in blue fond deviate from the respective upper limit for EVOO; PV: peroxide value

Table S3. Changes in FA composition (%FAMEs) of VOOs cv. 'Chondrolia Chalkidikis' and 'Chalkidiki' from the main olive mills of the regional unit of Chalkidiki (2016/17) during the 18-month storage in the dark at room temperature.

Samples	Storage time (months)	C16:0	C16:1	C17:0	C17:1	C18:0	C18:1	C18:2	C18:3	C20:0	C20:1	C22:0	C24:0
1	0	12.9	1.0	0.03	0.1	2.0	74.8	7.7	0.6	0.4	0.3	0.1	0.1
	6	12.4	1.1	0.04	0.1	2.1	75.1	7.6	0.6	0.4	0.4	0.1	0.1
	12	12.4	1.1	0.03	0.1	2.1	75.1	7.6	0.6	0.4	0.4	0.1	0.1
	18	12.4	1.1	0.03	0.1	2.1	75.2	7.6	0.6	0.4	0.4	0.1	0.1
2	0	13.2	1.0	0.04	0.1	2.1	75.0	6.8	0.7	0.5	0.4	0.1	0.1
	6	12.6	1.0	0.04	0.1	2.1	75.0	6.8	0.7	0.5	0.4	0.1	0.1
	12	12.6	1.0	0.04	0.1	2.1	75.7	6.7	0.7	0.4	0.4	0.1	0.1
	18	12.6	1.0	0.03	0.1	2.1	75.8	6.6	0.7	0.4	0.4	0.1	0.1
3	0	12.9	1.1	0.03	0.1	2.0	75.1	6.9	0.7	0.5	0.4	0.1	0.1
	6	12.9	1.1	0.03	0.1	2.0	75.1	6.9	0.7	0.4	0.4	0.1	0.1
	12	13.0	1.1	0.04	0.1	2.0	75.1	7.0	0.7	0.4	0.4	0.1	0.1
	18	12.8	1.1	0.04	0.1	2.0	75.4	6.9	0.7	0.4	0.3	0.1	0.1
4	0	14.2	1.1	0.04	0.1	1.8	74.2	6.9	0.7	0.4	0.3	0.1	0.1
	6	12.8	1.1	0.03	0.1	2.6	74.9	6.7	0.7	0.4	0.4	0.1	0.1

	12	13.2	1.1	0.03	0.1	2.0	75.1	6.7	0.7	0.4	0.4	0.1	0.1
	18	13.0	1.1	0.04	0.1	2.0	75.3	6.7	0.7	0.4	0.4	0.1	0.1
5	0	12.6	0.9	0.04	0.1	2.4	72.3	10.0	0.7	0.4	0.3	0.1	0.1
	6	11.9	0.9	0.03	0.1	2.5	72.9	10.0	0.7	0.4	0.3	0.1	0.1
	12	11.9	0.9	0.03	0.1	2.5	73.0	9.9	0.7	0.4	0.4	0.1	0.1
	18	12.1	0.9	0.03	0.1	2.5	72.8	9.9	0.7	0.4	0.3	0.1	0.1
6	0	12.5	1.0	0.04	0.1	2.3	74.9	7.7	0.6	0.4	0.3	0.1	0.1
	6	11.4	0.9	0.03	0.1	2.4	76.1	7.5	0.6	0.4	0.4	0.1	0.1
	12	11.5	0.9	0.03	0.1	2.2	76.1	7.6	0.6	0.4	0.4	0.1	0.1
	18	11.9	0.9	0.03	0.1	2.5	75.1	7.8	0.6	0.5	0.4	0.1	0.1
7	0	13.5	1.2	0.04	0.1	1.9	74.5	7.1	0.7	0.4	0.3	0.1	0.1
	6	13.0	1.2	0.03	0.1	2.0	75.0	7.1	0.7	0.4	0.4	0.1	0.1
	12	13.0	1.2	0.03	0.1	2.0	75.0	7.1	0.7	0.4	0.4	0.1	0.1
	18	13.0	1.2	0.03	0.1	2.0	75.1	7.1	0.7	0.4	0.4	0.1	0.1
8	0	13.7	1.2	0.04	0.1	2.0	74.4	7.4	0.7	0.4	0.3	0.1	0.1
	6	12.3	1.1	0.03	0.1	2.1	75.4	7.4	0.7	0.4	0.4	0.1	0.1
	12	12.3	1.1	0.03	0.1	2.0	75.4	7.3	0.6	0.4	0.4	0.1	0.1
	18	12.4	1.1	0.03	0.1	2.0	75.4	7.3	0.7	0.4	0.4	0.1	0.1

9	0	12.0	1.0	0.03	0.1	2.2	74.9	8.5	0.4	0.4	0.4	0.1	0.1
	6	11.5	1.1	0.03	0.1	2.2	75.2	8.5	0.6	0.4	0.4	0.1	0.1
	12	11.5	1.1	0.03	0.1	2.2	75.1	8.5	0.6	0.4	0.4	0.1	0.1
	18	11.5	1.0	0.02	0.1	2.2	75.2	8.4	0.6	0.4	0.4	0.1	0.1
10	0	11.8	0.9	0.04	0.1	2.3	74.1	9.3	0.6	0.4	0.3	0.1	0.1
	6	11.3	0.9	0.03	0.1	2.4	74.2	9.5	0.6	0.5	0.4	0.1	0.1
	12	11.2	0.9	0.03	0.1	2.4	74.3	9.5	0.6	0.5	0.4	0.1	0.1
	18	11.3	0.9	0.03	0.1	2.4	74.2	9.4	0.6	0.5	0.4	0.1	0.1
11	0	13.7	1.2	0.03	0.1	1.9	75.1	6.5	0.6	0.4	0.3	0.1	0.1
	6	12.4	0.8	0.04	0.1	3.0	73.2	8.8	0.7	0.5	0.3	0.1	0.1
	12	12.2	1.1	0.03	0.1	2.1	76.4	6.4	0.6	0.5	0.4	0.1	0.1
	18	12.2	1.1	0.03	0.1	2.1	76.4	6.5	0.6	0.5	0.4	0.1	0.1

Mean values (*n* = 2); fatty acids: palmitic, C16:0; palmitoleic, C16:1; heptadecanoic, C17:0; heptadecenoic, C17:1; stearic, C18:0; oleic, C18:1; linoleic, C18:2; linolenic, C18:3, arachidic, C20:0; gadoleic, C20:1; behenic,C22:0; Lignoceric acid, C24:0
